# Disruption of CXCR6 Ameliorates Kidney Inflammation and Fibrosis in Deoxycorticosterone Acetate/Salt Hypertension

**DOI:** 10.1038/s41598-019-56933-7

**Published:** 2020-01-10

**Authors:** Yuanbo Wu, Changlong An, Xiaogao Jin, Zhaoyong Hu, Yanlin Wang

**Affiliations:** 1 Section of Nephrology, Department of Medicine, Baylor College of Medicine, Houston, Texas USA; 2 Department of Anesthesiology, Hubei Cancer Hospital, Hubei Wuhan, China; 3 Division of Nephrology, Department of Medicine, University of Connecticut School of Medicine, Farmington, Connecticut USA; 4 Department of Cell Biology, University of Connecticut School of Medicine, Farmington, Connecticut USA; 5 Institute for Systems Genomics, University of Connecticut School of Medicine, Farmington, Connecticut USA; 6 Renal Section, Veterans Affairs Connecticut Healthcare System, West Haven, Connecticut USA

**Keywords:** Mechanisms of disease, Chemokines

## Abstract

Circulating cells have a pathogenic role in the development of hypertensive nephropathy. However, how these cells infiltrate into the kidney are not fully elucidated. In this study, we investigated the role of CXCR6 in deoxycorticosterone acetate (DOCA)/salt-induced inflammation and fibrosis of the kidney. Following uninephrectomy, wild-type and CXCR6 knockout mice were treated with DOCA/salt for 3 weeks. Blood pressure was similar between wild-type and CXCR6 knockout mice at baseline and after treatment with DOCA/salt. Wild-type mice develop significant kidney injury, proteinuria, and kidney fibrosis after three weeks of DOCA/salt treatment. CXCR6 deficiency ameliorated kidney injury, proteinuria, and kidney fibrosis following treatment with DOCA/salt. Moreover, CXCR6 deficiency inhibited accumulation of bone marrow–derived fibroblasts and myofibroblasts in the kidney following treatment with DOCA/salt. Furthermore, CXCR6 deficiency markedly reduced the number of macrophages and T cells in the kidney after DOCA/salt treatment. In summary, our results identify a critical role of CXCR6 in the development of inflammation and fibrosis of the kidney in salt-sensitive hypertension.

## Introduction

Chronic kidney disease is a growing public health problem, which is associated with increased cardiovascular morbidity and mortality and can progress to end stage kidney disease^1^. A major cause of chronic kidney disease is hypertension. Currently, therapeutic options for end-stage kidney disease are not effective except for dialysis or kidney transplantation^2^. Dialysis drastically reduces quality of like while kidney transplant is not without risks. To develop novel therapy for this devastating disorder, it is very important to dissect the molecular mechanisms causing the initiation and progression of chronic kidney disease.

Recent studies have implicated that circulating cells have a crucial role in the pathogenesis of hypertensive target-organ damage^3–6^. Chemokines and their respective receptors mediate the infiltration of circulating cells into injured sites^7^. Chemokine (C-X-C motif) ligand 16 (CXCL16) belongs to the CXC chemokine subfamily^8^. We have recently demonstrated that CXCL16 is upregulated in the kidney following deoxycorticosterone acetate (DOCA)/salt administration and genetic deletion of CXCL16 attenuates kidney injury and fibrosis in experimental model of DOCA/salt hypertension^9^. The receptor for CXCL16 is CXCR6, which is expressed on T cells, macrophages, and fibrocytes^10–12^. However, its role in kidney injury and fibrosis in DOCA/salt hypertension remains to be elucidated.

In this study, we used CXCR6 knockout (KO) mice to examine the role of CXCR6 in the development of kidney damage in DOCA/salt hypertension. Our results have demonstrated that genetic disruption of CXCR6 inhibits myeloid fibroblast accumulation and macrophage and T lymphocyte infiltration into the kidney, thereby suppressing kidney injury and fibrosis in DOCA/Salt hypertension.

## Results

### Disruption of CXCR6 does not affect blood pressure

**Figure 1:**
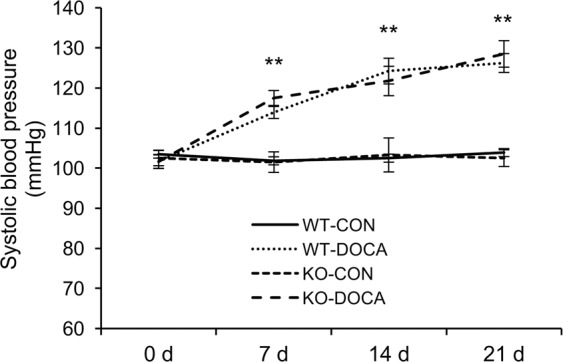
Disruption of CXCR6 does not affect blood pressure. **P < 0.01 between DOCA-salt groups and vehicle control groups.

After left nephrectomy, WT and CXCR6 KO mice received vehicle or DOCA/salt treatment for 3 weeks. At baseline, blood pressure was comparable among the four groups. Following DOCA/salt treatment, there was a significant increase in blood pressure. The increases in blood pressure were similar in both WT and CXCR6 KO mice (Fig. 1).

### Disruption of CXCR6 reduces albuminuria

**Figure 2:**
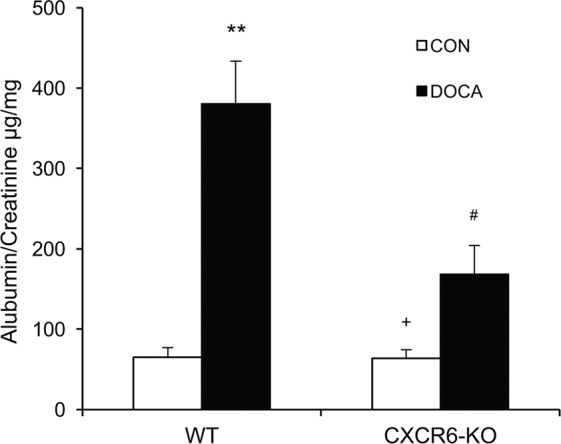
Disruption of CXCR6 reduces albuminuria in DOCA-salt hypertension. **P < 0.01 vs WT-CON, ^+^*P* < 0.05 vs KO-DOCA, ^#^*P* < 0.05 vs WT-DOCA. n = 6 in each group.

WT mice produced substantial albuminuria after treatment with DOCA/salt for three weeks in comparison with WT control mice as indicated by increased urine albumin to creatinine ratio. In contrast, CXCR6 KO mice developed significantly less albuminuria following three-week of DOCA/salt treatment (Fig. 2).

### Disruption of CXCR6 ameliorates kidney injury and fibrosis

**Figure 3:**
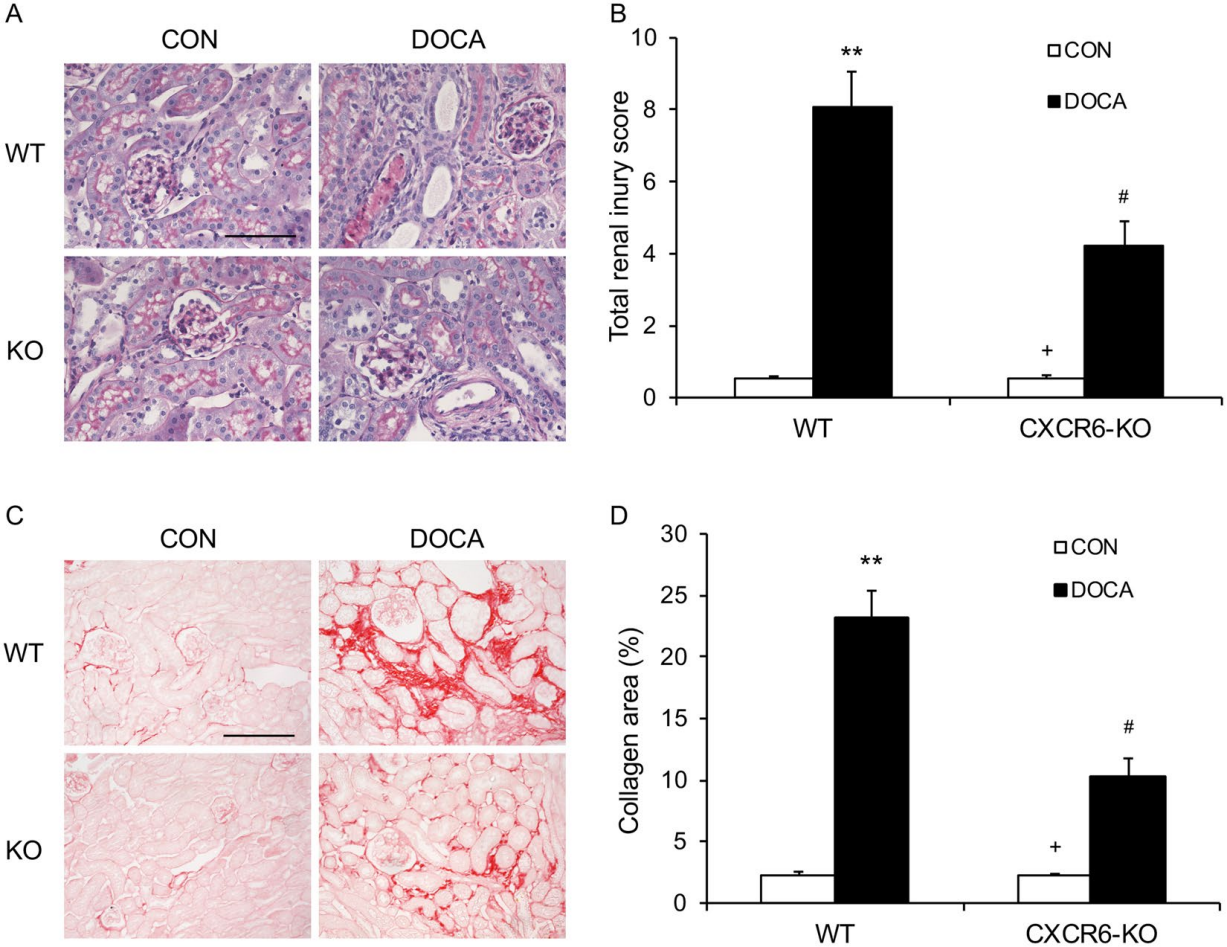
Disruption of CXCR6 attenuates renal injury and fibrosis. **(A**) Representative photomicrographs of periodic acid-Schiff-stained kidney sections. Scale bar, 50 μm. **(B**) Quantitative assessment of renal injury in WT and CXCR6 KO mice. **P < 0.01 vs WT-CON, ^+^*P* < 0.05 vs KO-DOCA, ^#^*P* < 0.05 vs WT-DOCA. n = 6 in each group. **(C**) Representative photomicrographs of kidney sections stained with sirius red for evaluation of total collagen deposition. Scale bar, 50 μm. **(D**) Quantitative analysis of interstitial collagen area in the kidneys. **P < 0.01 vs WT-CON, ^+^*P* < 0.05 vs KO-DOCA, ^#^*P* < 0.05 vs WT-DOCA. n = 6 in each group.

To examine the role of CXCR6 in the development of kidney damage, we stained the kidney sections with periodic acid-Schiff (PAS). There was minimal renal damage in the two vehicle-treated groups. WT mice displayed substantial kidney damage following treatment with DOCA/salt. Remarkably, CXCR6 deficient mice developed less severe kidney damage after DOCA/salt treatment (Fig. 3A,B).

To determine the role of CXCR6 in renal fibrosis, we stained the kidney sections with sirius red. Compared with vehicle-treated WT mice, DOCA/salt-treated WT mice displayed significant collagen deposition in the kidney. In contrast, CXCR6 deficient mice accumulated significantly less collagen in the kidney after treatment with DOCA/salt (Fig. 3C,D).

### Disruption of CXCR6 attenuates ECM protein expression

**Figure 4:**
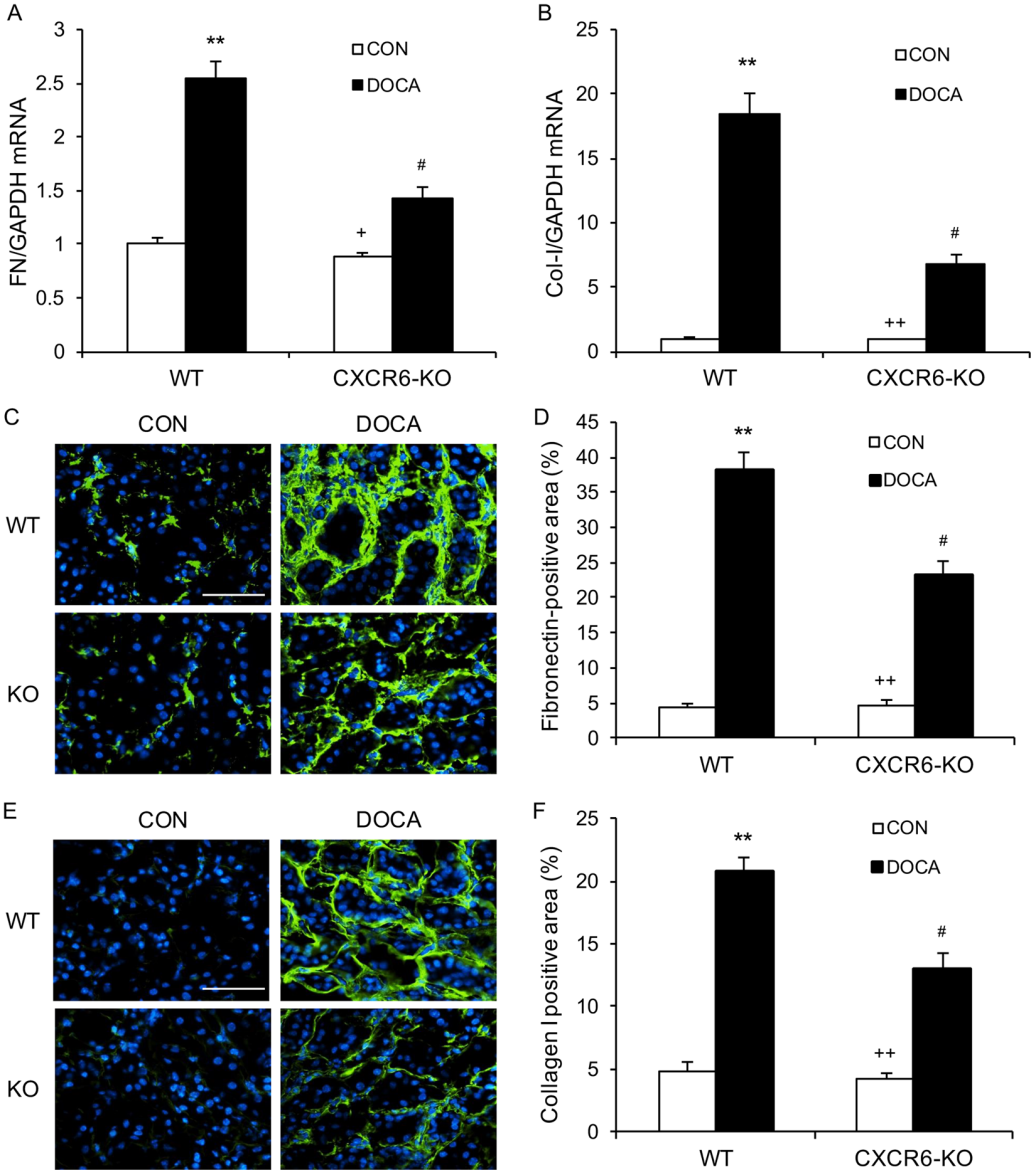
Disruption of CXCR6 inhibits fibronectin and collagen I expression. **(A**) The mRNA levels of fibronectin (FN) in the kidney of WT and CXCR6 KO mice in response to DOCA-salt. **P < 0.01 vs WT-CON, ^+^*P* < 0.05 vs KO-DOCA, and ^#^*P* < 0.05 vs WT-DOCA. n = 3–4 per group. **(B**) The mRNA levels of collagen I in the kidney of WT and CXCR6 KO mice in response to DOCA-salt. **P < 0.01 vs WT-CON, ^++^*P* < 0.01 vs KO-DOCA, and ^#^*P* < 0.05 vs WT-DOCA. n = 4 per group. (**C**) Representative photomicrographs of kidney sections stained for fibronectin (green) and counterstained with DAPI (blue). Scale bar, 50 μm. (**D**) Quantitative analysis of fibronectin-positive area in the kidneys. **P < 0.01 vs WT-CON, ^++^*P* < 0.01 vs KO-DOCA, ^#^*P* < 0.05 vs WT-DOCA. n = 6 in each group. (**E**) Representative photomicrographs of kidney sections stained for collagen I (green) and counterstained with DAPI (blue). Scale bar, 50 μm. (**F**) Quantitative analysis of collagen I-positive area in the kidneys. **P < 0.01 vs WT-CON, ^++^*P* < 0.01 vs KO-DOCA, ^#^*P* < 0.05 vs WT-DOCA. n = 6 in each group.

We next performed real-time RT-PCR to determine the mRNA levels of collagen I and fibronectin in the kidney. DOCA/salt treatment resulted in a substantial increase in the mRNA levels of fibronectin and type I collagen in the kidney of WT mice. In contrast, disruption of CXCR6 led to a significant reduction of the mRNA levels of fibronectin and type I collagen in the kidney after treatment with DOCA/salt (Fig. 4A,B). In agreement with these results, quantitative immunofluorescence analysis revealed that DOCA/salt-treated CXCR6 KO mice developed significant less deposition of fibronectin and type I collagen in the kidneys compared with DOCA/salt-treated WT mice (Fig. 4C–F).

### Disruption of CXCR6 inhibits myeloid fibroblasts accumulation

**Figure 5:**
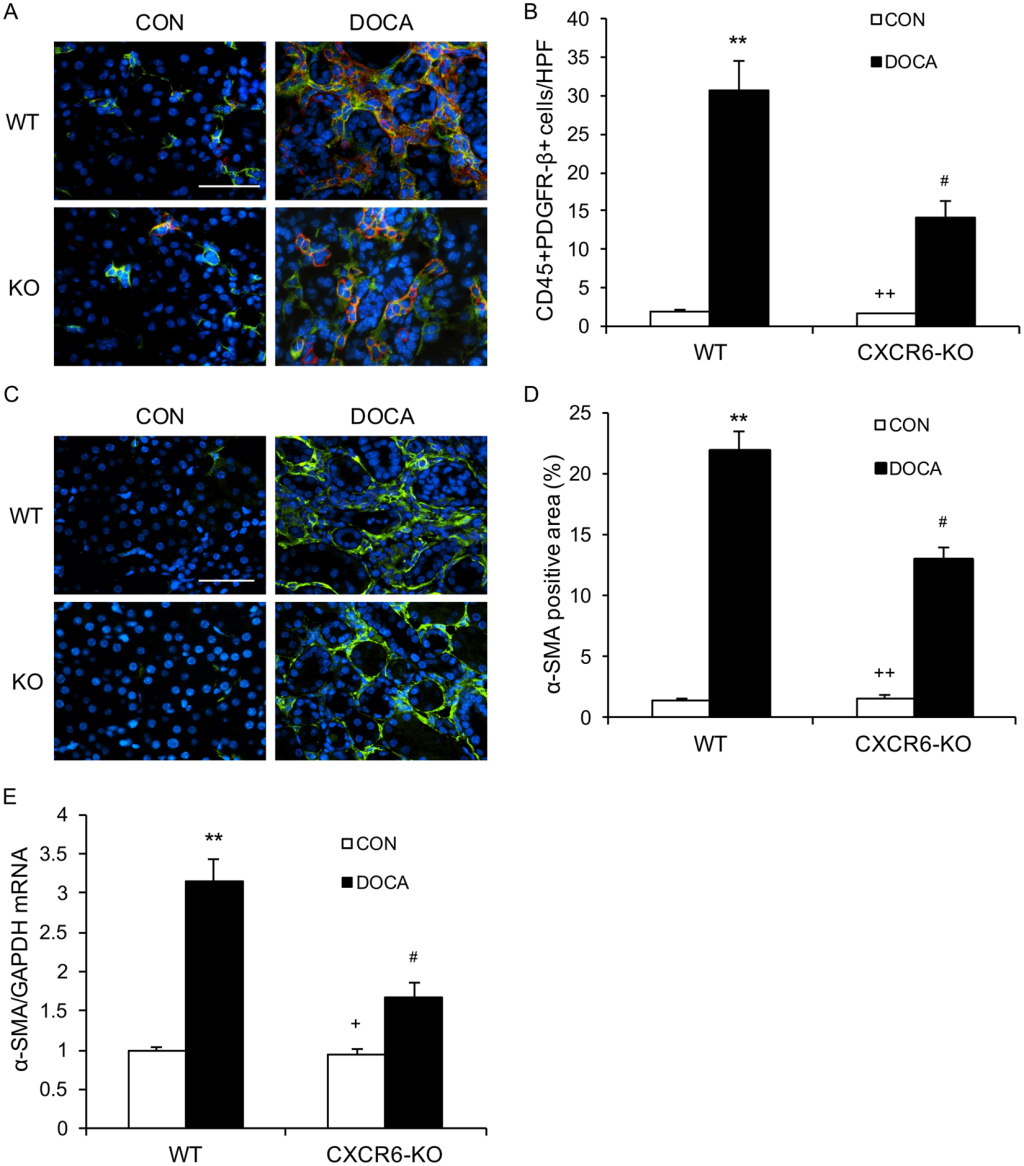
Disruption of CXCR6 suppresses bone marrow–derived fibroblast accumulation and myofibroblast formation in the kidney. (**A**) Representative photomicrographs of kidney sections stained for CD45 (red), PDGFR-β (green), and nuclei(DAPI; blue). Scale bar, 50 μm. (**B**) Quantitative analysis of CD45^+^ and PDGFR-β^+^ fibroblasts in kidneys. **P < 0.01 vs WT-CON, ^++^*P* < 0.01 vs KO-DOCA, ^#^*P* < 0.05 vs WT-DOCA. n = 6 in each group. (**C**) Representative photomicrographs of kidney sections stained for α-SMA. Scale bar, 50 μm. (**D**) Quantitative analysis of α-SMA positive area in kidneys. **P < 0.01 vs WT-CON, ^++^*P* < 0.01 vs KO-DOCA, ^#^*P* < 0.05 vs WT-DOCA. n = 6 in each group. (**E**) The mRNA levels of α-SMA in the kidney of WT and CXCR6 KO mice in response to DOCA-salt. **P < 0.01 vs WT-CON, ^+^*P* < 0.05 vs KO-DOCA, and ^#^*P* < 0.05 vs WT-DOCA. n = 4 per group. HPF, high-power field.

Myeloid fibroblasts are a major source of activated fibroblasts that are responsible for the development of renal fibrosis^12–14^. Myeloid fibroblasts express CXCR6^12^, thus, the role of CXCR6 in myeloid fibroblast accumulation in the kidney was assessed. We stained the kidney sections for CD45, a hematopoietic cell marker, and platelet-derived growth factor receptor β (PDGFR-β), a mesenchymal marker. WT mice accumulated a significant number of myeloid fibroblasts, positive for CD45 and PDGFR-β, in the kidney following DOCA/salt treatment. In contrast, CXCR6 deficient mice accrued fewer bone marrow–derived fibroblasts, positive for CD45 and PDGFR-β, in the kidney following treatment with DOCA/salt (Fig. 5A,B).

To examine the number of myofibroblasts in the kidney, we stained kidney sections for α-smooth muscle actin (α-SMA), a myofibroblast marker. WT mice accumulated a large number of myofibroblasts positive for α-SMA in the kidney after treatment with DOCA/salt for three weeks. CXCR6 deficiency significantly reduced the number of myofibroblasts positive for α-SMA in the kidney after treatment with DOCA/salt (Fig. 5C,D). In agreement with these findings, the mRNA levels of α-SMA were significantly decreased in the kidney of DOCA/salt-treated CXCR6 KO mice compared with DOCA/salt-treated WT mice (Fig. 5E). Likewise, the α-SMA protein levels were substantially reduced in the kidney of DOCA/salt-treated CXCR6 KO mice compared with DOCA/salt-treated WT mice (Fig. S1).

### Disruption of CXCR6 suppresses infiltration of macrophages and T lymphocytes into the kidney

**Figure 6:**
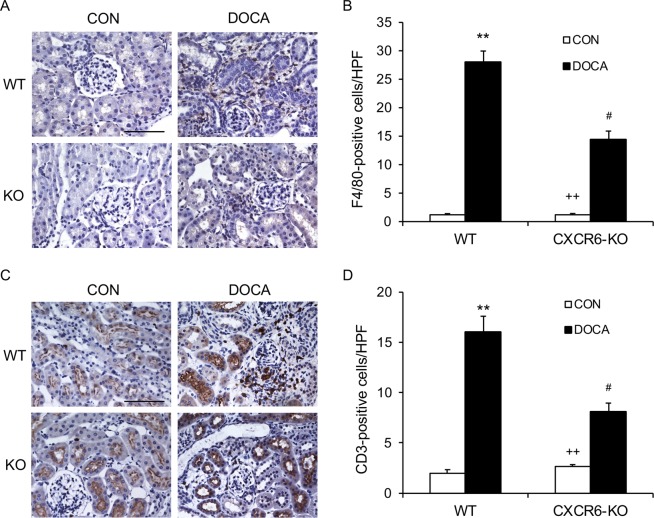
Disruption of CXCR6 inhibits infiltration of macrophages and T cells into the kidney. (**A**) Representative photomicrographs of kidney sections stained for F4/80 (brown) and counterstained with hematoxylin (blue). Scale bar, 50 μm. (**B**) Quantitative analysis of F4/80^+^ macrophages in kidneys. **P < 0.01 vs WT-CON, ^++^*P* < 0.01 vs KO-DOCA, ^#^*P* < 0.05 vs WT-DOCA. n = 6 in each group. (**C**) Representative photomicrographs of kidney sections stained for CD3 (brown) and counterstained with hematoxylin (blue). Scale bar, 50 μm. (**D**) Quantitative analysis of CD3^+^ T cells in kidneys. **P < 0.01 vs WT-CON, ^++^*P* < 0.01 vs KO-DOCA, ^#^*P* < 0.05 vs WT-DOCA. n = 6 in each group. HPF, high power field.

Macrophages and T lymphocytes participate in hypertension target organ damage^3,5,15^. To investigate if CXCR6 mediates macrophage and T cell infiltration into the kidney, we stained kidney sections for CD3, a T lymphocyte marker, and F4/80, a macrophage marker. DOCA/salt-treatment resulted in a significant increase in the numbers of macrophages and T lymphocytes in the kidney of WT mice. In contrast, disruption of CXCR6 substantially reduced the numbers of macrophages and T lymphocytes in the kidney after treatment with DOCA/salt for three weeks (Fig. 6).

## Discussion

In the present study, we examine the contribution of CXCR6 to the development of DOCA/salt-induced hypertensive nephropathy. The results of our study demonstrate that genetic disruption of CXCR6 protects the kidney against DOCA/salt-induced damage, inhibits myeloid fibroblast accumulation and activation, and suppresses macrophage and lymphocyte infiltration into the kidney in response to salt-sensitive hypertension induced by DOCA/salt. These results suggest that CXCR6 plays a critical role in the development of DOCA/salt-induced hypertensive nephropathy by mediating the recruitment of myeloid fibroblasts and inflammatory cells into the kidney.

Recently, we have demonstrated that CXCL16 recruits myeloid fibroblasts, macrophages and T lymphocytes into the kidney resulting in kidney injury and fibrosis in a DOCA/salt model of hypertension^9^. CXCL16 regulates circulating cell trafficking via interaction with its receptor CXCR6^16^. CXCR6 was initially cloned as an orphan receptor known as STRL33/BONZO/TYMSTR^17^. It has been reported that CXCR6 is expressed on T lymphocytes, monocytes/macrophages, and myeloid fibroblasts^10–12^. One study reports that CXCR6 mediates leukocyte trafficking into the liver resulting in hepatic fibrosis in experimental steatohepatitis^18^. Another study shows that the CXCL16/CXCR6 axis can activates phosphatidylinositide 3-kinase/Akt signaling to stimulate platelet activation and adhesion^19^. We have recently demonstrated that CXCR6 is involved in kidney inflammation and fibrosis in animal models of angiotensin II-induced hypertension and obstructive nephropathy^6,20^. In the present study, we utilized CXCR6 knockout mice to examine the role of CXCR6 in kidney damage in a salt-sensitive model of hypertension induced by DOCA/salt. Our results show that blood pressure is not affected by genetic disruption of CXCR6 under normal condition and following DOCA/salt administration. In contrast, urinary albumin excretion and glomerular and vascular damage of the kidney are significantly attenuated in CXCR6 deficient mice treated with DOCA/salt. These results support a crucial role of CXCR6 in DOCA/salt-induced hypertensive kidney damage.

Kidney fibrosis is a prominent pathological feature of hypertensive nephropathy. The severity of interstitial fibrosis correlates positively with the progression of chronic kidney disease^21^. In the present study, we have demonstrated that the degree of kidney fibrosis as indicated by sirius red staining is markedly reduced in CXCR6 deficient mice with DOCA/salt hypertension. Furthermore, the mRNA and protein levels of fibronectin and type I collagen in the kidney are significantly suppressed in CXCR6 deficient mice following treatment with DOCA/salt. These data suggest that the development of kidney fibrosis in DOCA/salt-induced hypertension is dependent on CXCR6.

Activated fibroblasts are the primary cells that are responsible for the production and deposition of ECM proteins during the pathogenesis of kidney fibrosis^22,23^. Recent studies reveal that myeloid fibroblasts contribute significantly to the population of activated fibroblasts^12,20,24,25^. Myeloid fibroblasts express hematopoietic markers such as CD45 and mesenchymal markers such as PDGFR-β^24,26^. In the present study, we have demonstrated that DOCA/salt treatment resulting in accumulation of myeloid fibroblasts and myofibroblasts in the kidney and development of fibrosis of the kidney of WT mice. Genetic disruption of CXCR6 reduces the number of myeloid fibroblasts and myofibroblasts in the kidney of mice treated with DOCA/salt. These results support a crucial role of CXCR6 in mediating the recruitment of myeloid fibroblasts into the kidney resulting in kidney fibrosis in salt-sensitive hypertension triggered by DOCA/salt.

Another salient feature of hypertensive kidney disease is inflammatory and immune cell infiltration^5,27–31^. Inflammatory and immune cells are recruited into the kidney by chemokines through interacting with their respective receptors^6,26,32^. Recently, we have demonstrated that genetic deletion of CXCL16 suppresses inflammatory and immune cell infiltration into the kidney following DOCA/salt-induced hypertension^9^. In the present study, we report that the infiltration of macrophage and T lymphocytes into the kidney is dramatically reduced in CXCR6 deficient mice treated with DOCA/salt. These findings suggest that CXCR6 mediates the trafficking of macrophages and T lymphocytes into the kidney following DOCA/salt-induced hypertension.

One potential limitation of our study is the method of measuring blood pressure. We utilized the BP-2000 Blood Pressure Analysis System™ to measure blood pressure. This blood pressure measurement system is a non-invasive method that does not require the anesthetization of the animal. In order to reduce variability, all data were collected during the same period of the day, by the same person, and under similar laboratory conditions. This is the most widely used method to measure blood pressure in rodents. Studies have shown that this method is very reliable and closely correlated to the intra-arterial blood pressure of mice since it was first introduced in 1995^33^. We have successfully used this method to measure blood pressure in mice as previously published^4,6,34^.

In summary, our study uncovers a critical role of CXCR6 in the development of kidney damage and fibrosis in DOCA/salt-induced hypertension. In DOCA/salt model of hypertension, CXCR6 functions as a crucial mediator in recruiting myeloid fibroblasts, macrophages and T lymphocytes into the kidney resulting in kidney damage and fibrosis. These results indicate that CXCR6 may be explored as a novel therapeutic strategy for hypertensive nephropathy.

## Material and Methods

### Animals

WT C57BL/6 mice and CXCR6 KO mice on C57BL/6 background were purchased from the Jackson Laboratory (Bar Harbor, ME). The expression of CXCR6 in the kidney of both WT and CXCR6 KO were confirmed as previously reported^6^. Mice were bred and maintained in a local animal care facility and had free access to food and water. Animal protocols were approved by the Baylor College of Medicine Institutional Animal Care and Usage Committee in agreement with PHS Policy on Humane Care and Use of Laboratory Animals and the US Animal Welfare Act (AWA).

### DOCA/salt model of hypertension

Mice of male gender at 8–10 weeks old were anesthetized with ketamine (80 mg/kg), xylazine (10 mg/kg), and acepromazine (3 mg/kg) through intraperitoneal injection. After removal of left kidney, mice were implanted with DOCA pellet (50 mg; Innovative Research of America, Sarasota, FL) subcutaneously in the neck region for three weeks^9^. DOCA-treated mice received 1% sodium chloride in drinking water. Control mice were subjected to left nephrectomy without DOCA pellet and 1% sodium chloride in drinking water. A total of 24 mice were used in the study with 6 mice in each group.

### Blood pressure

The BP-2000 blood pressure analysis system (Visitech Systems, Apex, NC) was used to measure systolic blood pressure (SBP) as previously described^4,6,9^.

### Albuminuria

Metabolic cages were used to collect urine one day before the end of experiments. Albumin and creatinine in the urine were measured as previously reported^4,6,9^.

### Histopathologic analysis

Kidney tissues were fixed in formalin, embedded in paraffin, and processed as previously described^4,6,9^. Kidney sections were stained with PAS to assess pathological abnormalities as described^9,35^, where 0 represented no abnormality and where 1, 2, 3, and 4 represented mild, moderate, moderately severe, and severe abnormalities respectively. Kidney sections were stained with sirius red to evaluate total collagen deposition in the kidney. Images of kidney sections were captured using a microscope equipped with a digital camera (Nikon Instruments Inc., Melville, NY), and quantitative analysis was performed in blinded fashion by one observer^4,6,12^. The sirius red-positive area was expressed as a percentage of the total area.

### Immunohistochemistry

Immunohistochemical staining was performed on paraffin sections as described^36,37^. After antigen retrieval, endogenous peroxidase activity was quenched with 3% hydrogen peroxide. The kidney sections were incubated with primary antibodies, appropriate secondary antibodies and ABC solution sequentially as described^9^. Immunoreactivity was detected with diaminobenzidine. Sections were counterstained with hematoxylin and mounted using histomount. A Nikon microscope image system (Nikon Instruments, Melville, NY) was used to capture the images and analyzed using a NIS Element software (Nikon Instruments, Melville, NY) in a blinded fashion by one observer^4,6^.

### Immunofluorescence

Immunofluorescence staining was performed according to an established protocol^9,24,25^. Kidney sections were fixed and stained with rabbit anti-collagen I antibody (Rockland Immunochemicals, Limerick, PA), rabbit anti-fibronectin antibody (Sigma-Aldrich, St. Louis, MO), or rabbit anti-α-SMA antibody (Abcam, Cambridge, MA) followed by Alexa-488 conjugated donkey anti-rabbit antibody (Invitrogen, Carlsbad, CA). For double immunofluorescence staining, kidney sections were fixed and stained with rat anti-CD45 (BD Biosciences, San Jose, CA) and platelet-derived growth factor receptor (PDGFR)-β (Santa Cruz Biotechnology, Dallas, TX) followed by appropriate secondary antibodies. Slides were mounted with medium containing DAPI. Fluorescence intensity was captured using a fluorescence microscope (Nikon Instruments Inc., Melville, NY). Quantitative evaluation was performed in a blinded manner by one observer using a NIS-Elements software. The fluorescence-positive area was reported as a percentage of the total high-power field (HPF) area^4,6,38^.

### Quantitative real-time RT-PCR

Quantitative analysis of the target mRNA expression was performed with real-time reverse transcription – polymerase chain reaction (RT-PCR) by the relative standard curve method^12^. Total RNA was extracted from kidney tissues with TRIzol Reagent (Invitrogen, Carlsbad, CA). Aliquots (1 µg) of total RNA were reverse-transcribed and amplified using IQ SYBR green supermix reagent (Bio-Rad, Herculus, CA). The expression levels of the target genes were normalized by the GAPDH level in each sample. The followings are the sequences of the primers. Collagen I: Forward 5′-TGCCGCGACCTCAAGATGTG-3′ and reverse 5′-CACAAGGGTGCTGTAGGTGA-3′; Fibronectin: Forward 5′-CTTCTCCGTGGAGTTTTACCG-3′ and reverse 5′- GCTGTCAAATTGAATGGTGGTG-3′; α-SMA: Forward 5′-ACTGGGACGACATGGAAAAG-3′ and reverse 5′-CATCTCCAGAGTCCAGCACA-3′; GAPDH: Forward 5′-TGCTGAGTATGTCGTGGAGTCTA-3′ and reverse 5′-AGTGGGAGTTGCTGTTGAAATC-3′.

### Statistical analysis

Data were expressed as mean ± SEM. Multiple group comparisons were performed by ANOVA followed by the Bonferroni procedure for comparison of means. A *P* value < 0.05 was considered statistically significant.

## Supplementary information

Supplementary Information
