# Supplementary material for: Disruption of CXCR6 Ameliorates Kidney Inflammation and Fibrosis in Deoxycorticosterone Acetate/Salt Hypertension
Source: Sci Rep. 2020 Jan 10;10:133. doi: 10.1038/s41598-019-56933-7 (PMC6954216; doi:10.1038/s41598-019-56933-7)

Disruption of CXCR6 Ameliorates Kidney Inflammation and Fibrosis in Deoxycorticosterone Acetate/Salt Hypertension

Supplemental Material

Yuanbo Wu[1,2], Changlong An[3], Xiaogao Jin[1], Zhaoyong Hu[1], and Yanlin Wang[1,3,4,5,6]*

[1] Section of Nephrology, Department of Medicine, Baylor College of Medicine, Houston, Texas, USA

[2] Department of Anesthesiology, Hubei Cancer Hospital, Hubei, Wuhan, China

[3] Division of Nephrology, Department of Medicine, University of Connecticut School of Medicine, Farmington, Connecticut, USA

[4] Department of Cell Biology, University of Connecticut School of Medicine, Farmington, Connecticut, USA

[5] Institute for Systems Genomics, University of Connecticut School of Medicine, Farmington, Connecticut, USA

[6] Renal Section, Veterans Affairs Connecticut Healthcare System, West Haven, Connecticut, USA

Running Title: CXCR6 in deoxycorticosterone acetate/salt hypertension

*Corresponding author:

Yanlin Wang, MD, PhD

Division of Nephrology/Department of Medicine

University of Connecticut School of Medicine

263 Farmington Avenue

Farmington, CT 06030-1405

Email: yanlwang@uchc.edu

Figure S1. Disruption of CXCR6 reduces α-SMA protein expression in the kidney. **A.** Representative Western blots show the protein levels of α-SMA in the kidneys of WT and CXCR6 KO mice 4 weeks after DOCA/salt or vehicle treatment. **B.** Quantitative Western blot analysis of α-SMA protein levels in the kidneys. ** *P*<0.01 vs WT controls, $^{+}$ *P*<0.05 vs KO-DOCA, and $^{\#}$ *P*<0.05 vs WT-DOCA. n=6 per group.

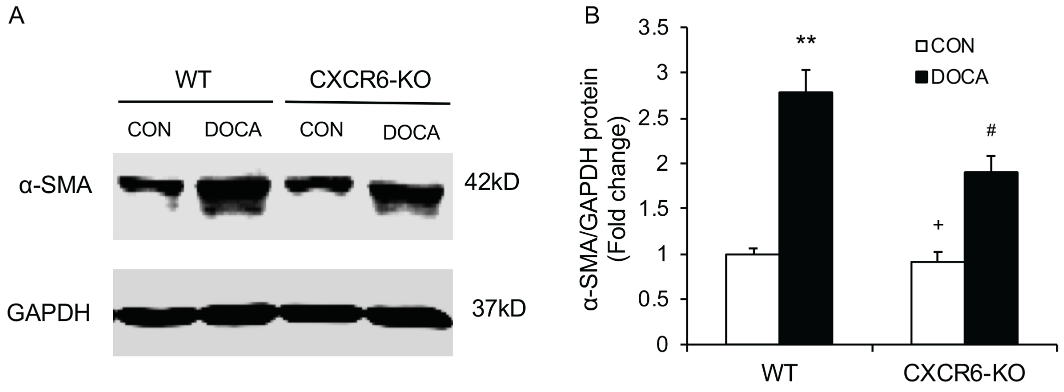

Supplement: Supplementary file 1 — Supplementary Information [file 41598_2019_56933_MOESM1_ESM.pdf]
